# Asymptomatic *Plasmodium falciparum* infections may not be shortened by acquired immunity

**DOI:** 10.1186/s12936-015-0813-1

**Published:** 2015-08-04

**Authors:** Michael T Bretscher, Nicolas Maire, Ingrid Felger, Seth Owusu-Agyei, Tom Smith

**Affiliations:** Swiss TPH, Basel, Switzerland; University of Basel, Basel, Switzerland; Kintampo Health Research Center, Kintampo, Ghana

**Keywords:** *Plasmodium falciparum*, Malaria, Immunity, Duration of infection, Mathematical model, Immigration-death model, Merozoite surface protein 2, Detectability, Cohort study

## Abstract

**Background:**

The duration of untreated *Plasmodium falciparum* infections is a defining characteristic of the parasite’s biology. It is not clear whether naturally acquired immunity (NAI) can shorten infections, despite the potential implications for malaria control and elimination as well as for basic research.

**Methods:**

Data on the presence of *P. falciparum msp2* genotypes in six blood samples collected over one year was analysed, together with four samples collected over 1 week, from a cohort in Navrongo (Ghana). Mathematical models assuming either exponential, Weibull, gamma, or log-normal infection durations were estimated separately for six age-groups. The method allowed for varying clonal acquisition and detection rates.

**Results:**

The best fitting (Weibull) mean durations were 124 days (children <5years old), 179 days (5–9 years), and 70–90 days (>10 years). This non-monotonic age pattern is not suggestive of an infection-clearing effect of NAI since immunity increases with exposure, and thus, age. Age-related differences in innate immunity are a more plausible explanation. 21% of blood-stage infections terminated within 1 week, in stark contrast to months of persistence in infections induced in neuro-syphilis patients (malariatherapy data). Age independence in this percentage raises the possibility that this clearance may result from innate mechanisms or genetic incompatibility between hosts and parasites, rather than from NAI.

**Conclusion:**

In all ages of hosts a substantial proportion of infections are cleared in the first days or weeks of appearance in the blood, while others persist for many months. Although cumulative exposure and NAI increase with age, this does apparently not translate into an increased rate of termination of infections.

**Electronic supplementary material:**

The online version of this article (doi:10.1186/s12936-015-0813-1) contains supplementary material, which is available to authorized users.

## Background

Malaria caused by *Plasmodium falciparum* continues to be among the top global public health problems. It causes an estimated 560,000 fatalities per year, mostly children under 5 years of age [1]. The parasite has many immune-evasion mechanisms: it displays extensive antigenic diversity, is able to switch between surface antigen variants, and several parasitic stages in the human host are poorly immunogenic [2]. How naturally acquired immunity (NAI) affects the duration of blood-stage infections is not well understood. It has been theorized that the duration of infections might be longer in relatively immune individuals, rather than shorter [3]. A better understanding of the epidemiological effects of NAI against malaria is warranted to guide efforts to control and potentially eliminate the disease. Moreover, knowledge of both effects and mechanisms of NAI is key to designing highly effective malaria vaccines.

Information on the effects of NAI can be obtained from (observational) epidemiological data by interpreting changing infection and disease patterns in response to exposure. Cumulative exposure (the total number of infections experienced by an individual) is proportional to host age for a given transmission intensity. This holds true even in the presence of spatial transmission heterogeneity. Consequently, age can be used as proxy for NAI. Analyses using this approach suggested that protection against non-cerebral severe malaria is acquired very quickly, after one or two infections [4]. NAI does not protect from re-infection with malaria, at least not completely, since many older individuals carry parasites even in areas of highest transmission intensity. Infections in such highly exposed individuals, however, are mostly asymptomatic, indicating a protective effect of NAI against clinical episodes [5]. Immune hosts generally have lower parasite counts and many of the clinical manifestations of malaria—fever, severe malaria, anaemia—can be directly related to high parasite densities [5, 6]. Therefore, the impact of NAI is most parsimoniously summarized as an improved ability to control parasite densities [5]. An important consequence of reduced parasite densities in immune individuals is reduced diagnostic sensitivity: parasites are hard to detect when present at low numbers, because too few parasites may be present in the blood sample analysed. This limitation not only affects microscopy but also methods based on Polymerase Chain Reaction (PCR), despite their substantially increased sensitivity. Here, the term “detectability” is used to describe the probability of detecting a particular infecting clone conditional on it being present in the host. This is similar to “sensitivity” but emphasizes host and parasite factors rather than the quality of a diagnostic test. The consequences of this detectability-reducing effect of NAI for the interpretation of epidemiological data are profound: it leads to uncertainty about whether exposure-related trends in malaria prevalence are due to reduction in acquisition of new infections, shorter infection durations, or merely due to lower detectability. Consequently, obtaining information on the effect of immunity on infection durations from epidemiological data is not straightforward. Historically this led to false confidence that an important effect of NAI is to significantly shorten *P. falciparum* infections[7]. For non-immune individuals, information on infection durations can be obtained from malaria therapy data. These data stem from patient records of the pre-antibiotic era, where malaria-induced fevers were used to treat neurosyphilis. The therapeutic infections lasted about 210 days on average and very short or very long durations were rare [8]. Malaria therapy data only contain information on infection durations in subjects with little or no previous exposure but remain the most important source of information on *P. falciparum* in-host dynamics. It is important to fill this knowledge gap by analyzing infection clearance in naturally exposed populations.

Multiple concurrent blood-stage infections within one human host are common in high-transmission settings since NAI does not protect from re-infection. How long the individual clones persist, how they interact with each other, and how their durations are affected by NAI is not well understood. Different infecting clones can be distinguished by genotyping parasite DNA from blood samples using highly polymorphic molecular markers. Provided high marker diversity in the parasite population the multiplicity of infection (MOI, number of co-infections) can be determined as the number of distinct PCR fragment sizes per sample. Age trends in such MOI measurements, however, do not directly reveal the effects of NAI on infection clearance—because clonal detectability itself declines with immunity [9]; this is analogous to the interpretation of age trends in microscopy prevalence explained above. To estimate duration from genotyping data, individual parasite clones must be tracked over time, in repeated samples (cohort studies). However, simply recording the time between first and last appearance of a genotype as estimate of infection duration gives false results: due to the low detectability it is impossible to tell for how long a genotype was already present before the first detection, and for how long it persisted after the last one. In extreme cases, start and endpoints of infection may well be before and after the study, respectively. Consequently, specialized statistical models must be used which simultaneously estimate force of infection, duration and detectability parameters. “Immigration-death” (ID) stochastic models [10] satisfy this requirement. To further gain insight on the effects of NAI on infection clearance, the data must comprise hosts of all ages, such that age trends can be interpreted. To the authors’ best knowledge only a single dataset is available which meets these criteria for investigating the effect of immunity on infection clearance: a cohort study from Navrongo, Northern Ghana, where individual clones were tracked longitudinally in two-monthly samples by identifying alleles at the highly polymorphic locus “Merozoite Surface Protein 2” (*msp2*) [11].

A number of articles have used the Navrongo dataset to assess the persistence of individual clones using statistical models that allow for imperfect parasite detection [9, 10, 12–15]. Of these, [15] and [9] are the most recent. Felger et al. [9] modelled infection clearance using exponential distributions, thereby estimating the mean duration of clonal infections separately for different age groups. The duration was found to be higher in 1–9 year old children (between 196 and 319 days) than in individuals below one and above 10 years (between 130 and 155 days). No decreasing trend with host age, and by implication exposure, was observed. Clonal detectability was found to decrease monotonically from about 50% in young children to approximately 10% in older adults. No decrease of the force of infection (FOI, no. of blood stage infections acquired per person per year) with host age was observed as would be expected if infection-blocking immunity were gradually building up over time [9]. Taken together, these results imply that (1) NAI does not decrease the average duration of clonal infections, (2) NAI does most likely reduce the clonal detectability, (3) no additional infection-blocking immunity is acquired compared to the youngest age group, even after decades of exposure, and (4) the observed age-decrease in PCR prevalence and MOI in the dataset must be caused by a decrease of detectability alone, because both FOI as well as duration (which together determine the proportion infected) do not decrease with age.

An important limitation of using an exponential distribution as model for infection clearance is that only the mean duration is estimated. No information can be obtained on whether—for example—all infections are cleared exactly on the day corresponding to the average duration or if the estimated average in fact arises from different subpopulations with short and long durations. The reason why most disease models assume exponential clearance of infections is because it simplifies the required mathematics enormously. A method for using Weibull, lognormal or gamma survival distributions ([15], Table 1) to analyse the Navrongo dataset was developed previously.

**Table 1:** Mathematical models for clearance of infections

Survival distribution	Scale	Shape	Mean	Variance	PDF	CDF
Exponential	$1/\mu > 0$	-	$1/\mu$	$1/\mu ^2$	$\mu e^{-\mu x}$	$1-e^{-\mu x}$
Weibull	$\lambda > 0$	$k > 0$	$\lambda \Gamma \left( 1+\frac{1}{k}\right)$	$\lambda ^2\Gamma \left( 1+\frac{2}{k}\right) - \mu ^2$	$\frac{k}{\lambda }\left( \frac{x}{\lambda }\right) ^{k-1}e^{-(x/\lambda )^{k}}$	$1- e^{-(x/\lambda )^k}$
Log-Normal	$\mu$	$\sigma > 0$	$e^{\mu +\sigma ^2/2}$	$(e^{\sigma ^2}-1) e^{2\mu +\sigma ^2}$	$\frac{1}{x \sigma \sqrt{2 \pi }}e^{-\frac{(\ln x - \mu )^2}{2\sigma ^2}}$	$\frac{1}{2} + \frac{1}{2} \mathrm {erf}\left[ \frac{\ln x-\mu }{\sigma \sqrt{2}}\right]$
Gamma	$\theta > 0$	$k>0$	$k\theta$	$k\theta ^2$	$x^{k-1} \frac{\exp {\left( -x/\theta \right) }}{\Gamma (k)\,\theta ^k}$	$\frac{\gamma (k, x/\theta )}{\Gamma (k)}$

Infection durations were modelled using parametric survival distributions. The exponential distribution is specified by a single scale parameter (the mean duration of infection). All the others have increased flexibility due to an additional shape parameter (distribution-specific parameter names are ignored). The following abbreviations are used in the table: for the gamma function $\Gamma (z) = \int _0^\infty t^{z-1} e^{-t}\,dt$, for the lower incomplete gamma function $\gamma (s,x) = \int _0^x t^{s-1}\,e^{-t}\,dt$, and for the error function ${\text {erf}}(x) = \frac{2}{\sqrt{\pi }}\int _0^x e^{-t^2} dt$.

**Fig. 1:**
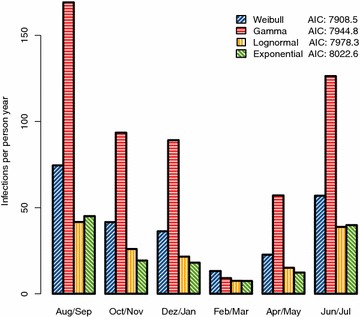
Seasonal Transmission. Each group of *bars* shows the FOI estimates for one 2-month season. Estimates differ considerably depending on the mathematical model for infection clearance, but agree with respect to the pattern of seasonality. The Weibull model clearly gave the best fit to the data, thus yielding the most reliable estimates.

The obtained estimates revealed a large proportion of very short infections, lasting only days or a few weeks. This is very different from malaria therapy data where most infections lasted several months if not treated. Also the average duration of blood-stage infections measured by [15] was lower (140 days) than in malariatherapy data (210 days) [8]. Transmission intensity is very high in Navrongo, estimated at 200–600 infectious mosquito bites per year [16]. Therefore, the abundance of very short infections and a shortened average duration in [15] may be interpreted as a saturation phenomenon at the blood-stage level.

The objective of the present analysis is to better understand the results of [15] in two ways. Firstly, more accurate information on the frequency and durations of short infections will be obtained by analyzing data with sampling intervals of one to five days. These samples, described in [17], were collected from the same hosts immediately after the two-month samples used by [9, 15]. Secondly, it will be investigated whether NAI can explain in the clearance of the shortest blood-stage infections, by estimating the distribution of infection durations separately for different age groups. Should immunity acquired over years of exposure be involved, then short infections are expected to be rare in young individuals and to increase with host age.

## Methods

The field study, conducted in the Kassena-Nankana district in the upper East region of Ghana was described previously [10, 14, 18, 19]. The malariological situation in this area is characterized by very high prevalence and multiplicity of infection [18, 20], and year-round transmission with seasonal variation in intensity [14].

### Study design and analysis methods

Blood was collected on ISOCodeStix™ PCR template preparation dipsticks (Schleicher & Schuell, Dassel, Germany). The study design comprised two distinct schemes for sample collection: firstly, a total of 349 individuals of all ages were visited every 2 months over the period of 1 year. Participants were chosen from randomly selected compounds in the study area, in an age-stratified manner [18]. This yielded the “long-interval” data, which was analysed using a previously described statistical method [15]. From the 349 participants of the main study, 80 individuals below 20 years of age were randomly selected for further sampling at shorter time intervals. Three additional blood samples were taken at day one, six and seven after the last survey of the main study. Study participants were visited in the early mornings of each day and houses were visited in the approximately the same order, to ensure sample collection at roughly the same time of day for each individual. These additional three samples in conjunction with the last sample from the main study constitute the “short-interval” data, which had been used previously for the measurement of detectability [17]. The present analysis aims to confirm the presence of short infections by taking advantage of the high time-resolution of these data.

### Genotyping

DNA was eluted from ISOCodeStix™ Stix and screened for presence of *P. falciparum* by nested *msp2* PCR. Processing of stix and PCR conditions have been described in detail before [21]. Alleles of the length-polymorphic genotyping marker *msp2* were distinguished by capillary electrophoresis and GeneMapper® software. An in-house generated software facilitated further analysis of GeneMapper® output and transformed the data into formats suitable for data management and statistical analysis.

### Analysis of short interval data

Data from those 69 participants out of 80 with a complete set of four samples were analysed. The statistical analysis considered the presence of a decreasing time trend in the probability of detection of an individual clone. Taking as reference the sample where a clonal infection first appeared, a data point was generated for every subsequent sample by noting whether the genotype was detected again or not. The probability of re-detection after *t* days was modelled as $p=q e^{-\mu t}$, with clearance parameter $\mu$ and detectability *q*. This can be interpreted as *q* multiplied by the probability that a clone is not yet after *t* days, as given by the exponential survivor function $e^{-\mu t}$. The parameters $\mu$ and *q* were estimated by fitting the linearized model $ln(q) - \mu t$ using the glm function in R, assuming a Bernoulli error model and a log link function. This model was compared via a likelihood-ratio test against a null model with time-invariant detection probability *q*.

### Analysis of long interval data

The six blood samples per person from the main study, obtained in intervals of 2 months, were analysed using immigration-death models which allowed for non-exponential distributions of infection durations, as described earlier [15]. In short: after preparation of the data into suitable format, statistical models which simulate acquisition, loss and detection of clonal infections were fitted. Corresponding to the three simulated processes, three sets of parameters were estimated: the FOI parameters $\lambda$, the scale and shape parameters of the distribution constituting the model for infection clearance (*r* and *s*, respectively), and the detectability parameters *q*.

#### Data preparation

Only data of those participants who were present at all six survey rounds of the main study were included in the analysis. This reduced the number of individuals in the dataset to 216. Failure or success to detect a genotype was denoted by 0 or 1, respectively, giving rise to 63 possible types of longitudinal patterns of detection, containing at least one positive test result. These were numbered from one to 63 using their binary value (e.g., 000010 is pattern 2), and counted. Thus, for every host, a frequency distribution of binary patterns was obtained. Statistical models were formulated to predict the absolute frequency of each pattern, taking into account host age and seasonal transmission.

#### Immigration-death models

New parameterizations of previously described immigration-death models were fitted to the long-interval data [15]. These models simulate acquisition (since birth), loss and detection of clonal infections, using a FOI parameter $\lambda$, shape (*s*) and scale (*r*) parameters of a distribution of infection durations, and a probability of detection, *q*. Given model equations for these basic parameters of infection dynamics, the expected frequency distribution of binary patterns in a given host can be predicted, and a likelihood can be calculated. The following model parameterizations were used: for the time-dependent FOI parameter $\lambda (t)$ it was assumed that a pattern of seasonal transmission had repeated since the birth of every host, with the FOI being independent of host age. Consequently, the model estimated six seasonal FOI parameters, each one corresponding to the acting FOI between two survey rounds.

Clearance of infections was modelled using four parametric survival distributions: the exponential, Weibull, gamma and lognormal distributions (Table 1). These represent competing hypotheses about infection clearance, compared with respect to goodness of fit. The exponential distribution is characterized by a single scale parameter *r*, equal to the mean duration of a clonal infection. It does therefore not have any flexibility to yield detailed information on the properties of the clearance process. Nevertheless it has been widely used in infectious disease models, mainly due to it’s mathematical simplicity (assuming a constant clearance rate per time implies an exponential distribution of infection durations and makes it possible to ignore the age structure of the within-host infection population). The Weibull, lognormal and gamma distributions are characterized by two parameters, *r* and *s*. In the following these will be called “scale” and “shape” parameters, ignoring possible distribution-specific names. A separate set of scale and shape parameters was estimated for each of the following age groups (in years): $<$5, 5–9, 10–19, 20–39, 40–59, $\ge$60. This extension of previous analyses [15], which estimated a single parameter set over all ages, serves the purpose of investigating a dependence of infection clearance on previous exposure, and by implication, acquired immunity. The detectability parameter *q* was modelled as a logit-linear function of host age. For a host of given age *a* (in units of two months) the detectability can be calculated as $q(a)=\text{ logit }^{-1}(q_0 + q_1(a-\bar{a}))$, with average age $\bar{a}=120.72$ and estimated parameters $q_0$ and $q_1$. A detailed description of the statistical methods was given previously, and all models were implemented in the Java ™ language and fitted to the data by maximum likelihood, as described in [15].

## Results

Two sets of longitudinal *msp2* genotyping data were analysed using mathematical models that allow for imperfect detection of parasites. Both data sets stem from the same cohort study, but samples were collected at different time intervals. The “long-interval data”, with two-monthly sample collection and age-stratified study population, was analysed using non-exponential ID models as developed in [15]. This yielded estimates of the rates of acquisition, clearance, and detection of parasite clones, and how these parameters are influenced by host age, a proxy for immunity. The “short-interval data” comprised four samples collected within one week from individuals below 20 years (median age 5.2 years, inter-quartile range 3.5–9.7) [17]. This permitted a detailed investigation of the shortest infections in moderately exposed individuals of the Navrongo population.

### Parasite acquisition, clearance and detection in the long-interval data

A comprehensive epidemiological description of the long-interval genotyping data was given in [9]. The average prevalence of *P. falciparum* was 48% by microscopy and 75% by PCR. Positivity by PCR showed a peak in the 5–9 year old children with 93% and declined to 54% in individuals older than 60 years. Mean multiplicity measured in the PCR positive samples also peaked in the age group of 5–9 years at 6.9 and was lowest in the 60+ age group at 2.8 distinct alleles per person. A total of 103 different *msp2* genotypes were found, the most frequent genotype representing 10.2% of all fragments detected. In the present analysis of the long-interval data, four ID models, using distinct parametric survival distributions to describe clearance of clonal infections, were compared using Akaike’s Information Criterion (AIC) as a measure of goodness of fit (Tables 2, 3): the Weibull model very clearly fitted the data best (AIC: 7908.5), followed by the gamma (AIC: 7944.8), lognormal (AIC: 7978.3), and exponential (AIC: 8022.6) models. The AIC values can be directly compared to those in [15] since the same dataset and methodology were used. The overall pattern of transmission seasonality was consistent among all models of infection survival, but differences exist in the numerical estimates of the FOI (Fig. 1; Table 3). Estimates of the Weibull model ranged from 74.5 infections per person year in August and September to 13.2 infections in February and March, while the corresponding results from the exponential model were 45.1 and 7.4, respectively. The gamma model estimated a considerably higher FOI of 169.1 in the high-transmission season, but the FOI estimate for the low-transmission season (9.1) agreed well with the other models. The raw parameter estimates describing a distribution of infection durations cannot easily be interpreted directly. Therefore, the mean duration is reported. It can be calculated from the scale and shape parameters (Table 3) and distribution-specific expressions (Table 1). All models suggested an intermediate mean duration of infection in the youngest age group, a peak in the 5–9 year olds and a subsequent decrease of duration with increasing host age (cumulative exposure).

**Table 2:** Raw parameter estimates: force of infection and detectability

Survival model	FOI by season (person^-1^ year^-1^)						Detectability		AIC
	$\lambda _1$	$\lambda _2$	$\lambda _3$	$\lambda _4$	$\lambda _5$	$\lambda _6$	$q_0$	$q_1$ (2-months^-1^)	
Weibull	74.5	41.6	36.3	13.2	22.7	56.9	−0.72	−0.003727	7,908.5
Gamma	169.1	93.5	89.2	9.1	57.1	126.3	−0.84	−0.004692	7,944.8
Lognormal	41.7	26.0	21.6	7.5	15.1	38.8	−0.89	−0.004985	7,978.3
Exponential	45.1	19.3	18.1	7.4	12.3	39.8	−0.84	−0.002865	8,022.6

Separate FOI parameters ($\lambda _1, \lambda _2, \ldots, \lambda _6$) were estimated for each of the six 2-month seasons. For a host of given age *a* (in units of two months) the detectability can be calculated as $q(a)=\text{ logit }^{-1}(q_0 + q_1(a-120.72))$. Lower AIC values indicate a better fit to the data.

**Table 3:** Raw parameter estimates: clearance of infections

Survival model	Scale						Shape						AIC
	$r_1$	$r_2$	$r_3$	$r_4$	$r_5$	$r_6$	$s_1$	$s_2$	$s_3$	$s_4$	$s_5$	$s_6$	
Weibull	1.00	1.31	1.01	0.75	0.94	1.23	0.49	0.47	0.67	0.58	0.66	0.75	7,908.5
Gamma	9.02	12.37	6.24	6.64	7.06	8.44	0.10	0.10	0.12	0.09	0.10	0.12	7,944.8
Lognormal	1.68	1.86	1.54	1.51	1.62	1.71	1.32	1.37	1.06	1.06	1.18	1.24	7,978.3
Exponential	3.73	5.30	2.93	2.15	2.10	2.18	–	–	–	–	–	–	8,022.6

Parameter estimates are shown for all models and host age groups. The exponential distribution is defined by a single “scale” parameter per age group, while all other distributions require an additional “shape” parameter. Derived measures, such as the mean duration, are obtained in conjunction with the equations in Table 1. The survey interval of two months was used as time unit where applicable. Lower AIC values indicate a better fit to the data.

**Table 4:** Estimated mean duration of infections

Survival model	<5 years old	5–9 years old	10–19 years old	20–39 years old	40–59 years old	>60 years old	AIC
Weibull	124.2	178.8	80.2	70.2	75.3	87.7	7,908.5
Gamma	54.6	77.4	43.1	36.8	40.6	59.7	7,944.8
Lognormal	241.9	285.5	161.7	158.4	194.6	220.4	7,978.3
Exponential	224.1	317.9	175.6	128.8	126.3	131.4	8,022.6

The mean duration of infection (in days) is shown for all age groups and clearance models. It can be calculated from the parameter estimates (Table 3) and the distribution-specific expressions for the mean (Table 1). Lower AIC values indicate a better fit to the data.

**Fig. 2:**
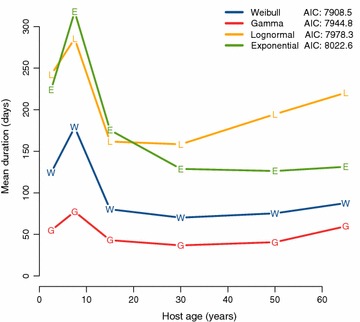
Effects of host age on the average infection duration. The average duration of clonal infections is shown against the midpoint of each age group and compared across clearance models. Lower AIC values indicate a better fit to the data. All models qualitatively agree with respect to the age pattern: an initial increase in duration during childhood, a sudden drop around the time of puberty, and no further decrease thereafter. The best-fitting Weibull model estimates that average duration remains constant in adults at ca. 80 days, even after decade-long exposure. Both the non-monotonic changes during childhood and adolescence as well as the absence of a change in adults are consistent with NAI not acting to shorten infections.

**Fig. 3:**
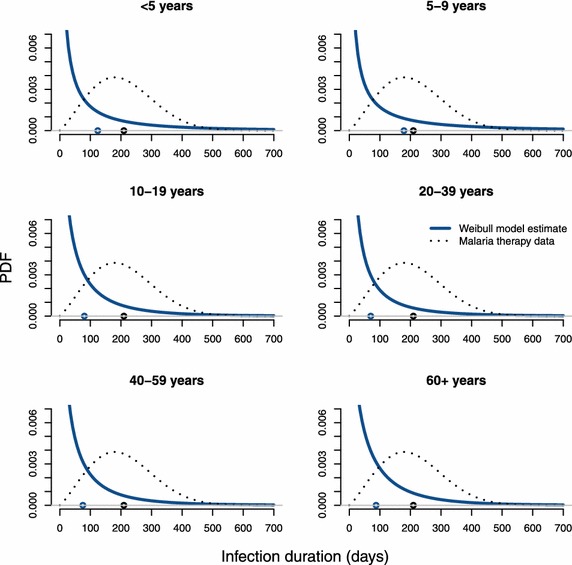
Short infections are similarly common in all age groups. The distribution of infection durations in Navrongo as estimated by the best-fitting Weibull model is illustrated for all age groups separately. Short infections are similarly common in all age groups, as indicated by the left-skewed PDF. An estimate from non-immune adult malaria therapy patients, where short infections are rare, is shown for comparison [8]. Mean durations are indicated by *circles* on the abscissa. If infections were cleared shortly after inoculation because of host immunity against particular antigenic variants one would expect an increase in the proportion of short infections with host age, a proxy for cumulative exposure.

A comparison of the best-fitting Weibull model with its age-independent counterpart in [15] found this age-pattern to be significant (p < 0.0001, LR-test). The average durations across all age groups were (in days): 102.7 (Weibull), 52.0 (gamma), 210.4 (lognormal), and 184.0 (exponential). Results for all age groups separately are shown in Table 4 and Fig. 2. All models suggested that in all age groups a significant proportion of infections was cleared shortly after appearance in the blood, as indicated by a strong positive skew of the distributions of infection duration (see Additional file 1). The probability density functions (PDF) of the best-fitting Weibull model are shown in Fig. 3 for all age groups, alongside the PDF estimated from non-immune malaria therapy patients for comparison [8]. The exponential distribution, which is commonly used in disease models due to its simplicity, depends by definition entirely on the mean duration and does not have the flexibility to measure the proportion of short infections. It fitted the data less well than the other models.

**Fig. 4:**
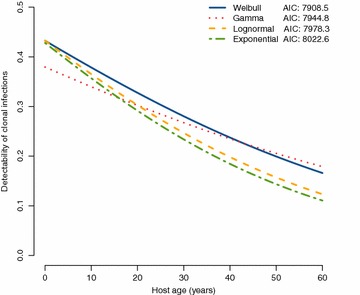
Effects of host age on clonal detectability. Detectability estimates from the long-interval data are in good agreement across different mathematical models. Starting from ca. 40% in children, the detectability of a single clone by PCR decreases with age to ca. 20%. Lower detectability in older hosts is likely due to a parasite density-reducing effect of NAI.

The estimates of detectability are in good quantitative agreement. All models measured a clonal detectability of ca. 40% in the young ages, which decreases to ca 20% in the older age groups (Fig. 4).

### Clearance of the shortest infections

**Fig. 5:**
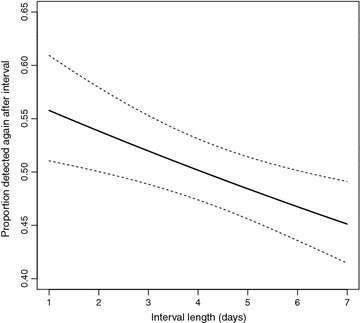
The probability of re-detecting clonal infections decreases with time. Shown is the probability that a genotype present in the first of two samples will also be detected in the second, together with a 95% confidence interval. This probability decreases with length of the time interval between surveys (p $<$ 0.001), at a rate corresponding to 3% of infections cleared per day, or 21% per week. This estimate was obtained from the short-interval data which permits tracking of clones at high time resolution, with four blood samples taken within 7 days.

In the short-interval data, the average prevalence across all four survey rounds was 46% by microscopy and 69% by PCR [17]. The dataset comprised 69 parasite-positive individuals between 6 months and 20 years of age, with a median age of 5.2 years (inter-quartile range 3.5–9.7). The median MOI among PCR-positives was 4. The 519 detected clones belonged to 77 different *msp2* genotypes, with the most common allele reaching a frequency of 9.2%. Fitting of the model $p=q e^{-\mu t}$, which describes the probability of re-detection of a genotype after time *t*, yielded an estimate for detectability at 0.58 as well as an estimated clearance rate of 0.0033 per day (p $<$ 0.001, LR test). This corresponds to 3% of infections being cleared per day, or 21% per week (Fig. 5).

## Discussion

The Navrongo long-interval data used in this work is unique in that it combines an age-stratified study design with high-resolution genotyping data and longitudinal follow-up of individuals. These features make it possible to gain detailed insight into the clearance of natural blood-stage infections, how this changes with host age, and how it compares to infection clearance in the non-immune, singly infected malariatherapy patients. Consequently, the Navrongo data has been analysed with progressively more complex mathematical models [9, 10, 14, 15]. The current analysis for the first time allows for non-exponential distributions of clearance times, permitting these distributions to vary with host age. The estimation of age-specific distribution parameters makes it possible to assess the effects of increasing exposure (implicitly a proxy for NAI). Previous analyses were restricted to either non-exponential clearance [15] or age trends [9]. The best model fit, both in the present analysis as well as in comparison to all previous ID models in [9, 14, 15], was obtained with Weibull-distributed clearance times and separate parameters for each age group. According to this model, the average persistence of individual clones peaked in 5–9 year olds, declined sharply after that, and did not further decrease with age (Fig. 2; Table 4). This statistically significant pattern is consistent with NAI not having an effect on infection duration. Durations first increased with age in young children despite an increase in the cumulative number of experienced infections with age. Similarly, decades of exposure in adults did not shorten infections any further. The non-monotonic age pattern of average infection duration may better be understood as a consequence of age-related changes in the immune system rather than as an effect of cumulative exposure. It is known that parasite densities are controlled more stringently with increasing host age [22]. This is due to age-related changes in the immune system rather than as a consequence of exposure, and similar factors may influence the duration of infection. It appears that the age group from 5–9 years should contribute more than others to the maintenance of malaria in regions with extended dry seasons, with their longer-lasting infections having a higher chance of persisting to the next transmission season.

The historical malaria therapy data are still the best source of data on the course of single untreated *P. falciparum* infections due to the ethical limitations on obtaining new, comparable data. Therefore it is necessary to discuss the present results in comparison to malaria therapy data despite caveats of how representative those may be for malaria in general. Two major differences between infection durations in the Navrongo residents compared to the malaria therapy patients were reported previously: a shorter average infection duration, and a much higher frequency of very short infections [15]. The present analysis of the Navrongo short interval data yielded more accurate information on the latter. It suggested that about one in five blood-stage infections in Navrongo were cleared within a week of appearance in the blood (Fig. 5). In contrast, few infections in the non-immune malaria therapy patients were cleared during the first weeks. Rather, the distribution of infection durations was approximately symmetrical about the mean [8, 23]. The somewhat shorter average duration in Navrongo together with the high frequency of very short durations consistently point towards increased clearance during the early phase of a blood-stage infection (in the sense that a proportion of short infections may explain a shorter average together with a second group of infections that last as long as those in the malaria therapy patients). It was hypothesized that this could be due to differences in immune status between patients and Navrongo residents [15]. According to this view, the highly exposed individuals in Navrongo might be immune against previously encountered genotypes, rapidly clearing these parasites with known antigenic profile from the blood. However, all ID models, including the best-fitting Weibull model, estimated that infections of short duration are similarly frequent in all age groups in Navrongo. This means that neither immunity acquired through years of exposure nor intrinsic age dependency in host responses can be important clearance promoting factors. An involvement of NAI would imply that short infections should become progressively more frequent in the older age groups. Provided, of course, that children in the youngest age group have not yet acquired infection-clearing immunity against all antigenic variants in circulation, which appears rather unlikely.

While a simple relationship between NAI and infection clearance is not apparent in the present results, it is difficult to identify the reasons behind the differences in infection clearance between Navrongo residents and malaria therapy patients. The shorter average duration in Navrongo together with the high frequency of short infections in all age groups is consistent with clearance by innate (non-specific) mechanisms which are independent of cumulative exposure. Almost all parasites in Navrongo meet the human host already occupied upon inoculation, while the malaria therapy patients were singly infected. A plausible cause for the rapid infection clearance in Navrongo may therefore be cross-reactive, transient host responses stimulated by high densities of one clone but acting against all co-infections [24]. Several experiments in non-falciparum animal models show that pre-existing infections can block the acquisition of new clones [25–27]. In humans, protection from clinical episodes as well as from superinfection due to pre-existing *P. falciparum* infections in humans has been documented [28–31]. Confirmation of this hypothesis, however, may require data where durations in singly and multiply infected hosts could be compared, perhaps from areas with lower transmission intensity or after radical parasite cure. Without such additional information, several alternative explanations cannot be excluded with certainty. Host- or parasite genetics could play a role [32] or differences in infective route and dose (some malaria therapy patients were infected using sporozoites, either through mosquito bites or via subcutaneous injection, and others through infected blood [8, 33]). One can be confident, however, that the frequency of short infections does not indicate unreported treatment among Navrongo residents: older individuals in such highly endemic areas are rarely symptomatic and consequently do not self-treat. One would thus expect short infections to be common among children, but very rare among adults.

The finding that cumulative exposure does not have an easily identifiable effect on infection clearance is somewhat counterintuitive. It appears plausible that the parasite may have evolved a strategy to prevent the human immune system from getting better at clearing infections with cumulative exposure. The difficulty in explaining the patterns of infection clearance in Navrongo illustrates the incompleteness of the scientific understanding of malaria in-host dynamics in naturally exposed populations.

## Additional file

Additional file 1: This document contains additional interpretations, analyses and figures based on the results of fitting all four candidate immigration-death models to the long-interval data. The implications of the estimated distributions of infection duration on the time to elimination, on the variation in infection durations and on senescence of infections are briefly discussed.

## References

[1] WHO (2013) World Malaria Report. http://www.who.int/malaria/publications/world_malaria_report_2013/en/. Accessed 31 July 2015

[2] Struik SS, Riley EM (2004). Does malaria suffer from lack of memory?. Immunol Rev.

[3] Recker M, Nee S, Bull PC, Kinyanjui S, Marsh K, Newbold C (2004). Transient cross-reactive immune responses can orchestrate antigenic variation in malaria. Nature.

[4] Gupta S, Snow RW, Donnelly CA, Marsh K, Newbold C (1999). Immunity to non-cerebral severe malaria is acquired after one or two infections. Nat Med.

[5] Doolan DL, Dobaáo C, Baird JK (2009). Acquired immunity to malaria. Clin Microbiol Rev.

[6] Müller I, Genton B, Rare L, Kiniboro B, Kastens W, Zimmerman P (2009). Three different Plasmodium species show similar patterns of clinical tolerance of malaria infection. Malar J.

[7] Sama W, Killeen G, Smith T (2004). Estimating the duration of *Plasmodium Falciparum* infection from trials of indoor residual spraying. Am J Trop Med Hyg.

[8] Sama W, Dietz K, Smith T (2006). Distribution of survival times of deliberate *Plasmodium falciparum* infections in tertiary syphilis patients. Trans R Soc Trop Med Hyg.

[9] Felger I, Maire M, Bretscher MT, Falk N, Tiaden A, Sama W (2012). The dynamics of natural *Plasmodium falciparum* infections. PLoS One.

[10] Sama W, Owusu-Agyei S, Felger I, Vounatsou P, Smith T (2005). An immigration-death model to estimate the duration of malaria infection when detectability of the parasite is imperfect. Stat Med.

[11] Smythe JA, Peterson M, Coppel RL, Saul AJ, Kemp DJ, Anders RF (1990). Structural diversity in the 45-kilodalton merozoite surface antigen of *Plasmodium falciparum*. Mol Biochem Parasitol.

[12] Smith T, Felger I, Fraser-Hurt N, Beck H (1999). Effect of insecticide-treated bed nets on the dynamics of multiple *Plasmodium falciparum* infections. Trans R Soc Trop Med Hyg.

[13] Smith T, Vounatsou P (2003). Estimation of infection and recovery rates for highly polymorphic parasites when detectability is imperfect, using hidden Markov models. Stat Med.

[14] Sama W, Owusu-Agyei S, Felger I, Dietz K, Smith T (2006). Age and seasonal variation in the transition rates and detectability of *Plasmodium falciparum* malaria. Parasitology.

[15] Bretscher MT, Maire N, Chitnis N, Felger I, Owusu-Agyei S, Smith T (2011). The distribution of *Plasmodium falciparum* infection durations. Epidemics.

[16] Appawu M, Owusu-Agyei S, Dadzie S, Asoala V, Anto F, Koram K (2004). Malaria transmission dynamics at a site in northern Ghana proposed for testing malaria vaccines. Trop Med Int Health TM IH.

[17] Bretscher MT, Valsangiacomo F, Owusu-Agyei S, Penny MA, Felger I, Smith T (2010). Detectability of *Plasmodium falciparum* clones. Malar J.

[18] Owusu-Agyei S, Smith T, Beck H, Amenga-Etego L, Felger I (2002). Molecular epidemiology of *Plasmodium falciparum* infections among asymptomatic inhabitants of a holoendemic malarious area in northern Ghana. Trop Med Int Health.

[19] Falk N, Maire N, Sama W, Owusu-Agyei S, Smith T, Beck H (2006). Comparison of PCR-RFLP and Genescan-based genotyping for analyzing infection dynamics of *Plasmodium falciparum*. Am J Trop Med Hyg.

[20] Binka F, Morris S, Ross D, Arthur P, Aryeetey M (1994). Patterns of malaria morbidity and mortality in children in northern Ghana. Trans R Soc Trop Med Hyg.

[21] Felger I, Irion A, Steiger S, Beck H (1999). Genotypes of merozoite surface protein 2 of *Plasmodium falciparum* in Tanzania. Trans R Soc Trop Med Hyg.

[22] Baird JK (1998). Age-dependent characteristics of protection v. susceptibility to *Plasmodium falciparum*. Ann Trop Med Parasitol.

[23] Collins WE, Jeffery GM (1999). A retrospective examination of sporozoite- and trophozoite-induced infections with *Plasmodium falciparum*: development of parasitologic and clinical immunity during primary infection. Am J Trop Med Hyg.

[24] Read AF, Taylor LH (2001). The ecology of genetically diverse infections. Science.

[25] de Roode JC, Helinski MEH, Anwar MA, Read AF (2005). Dynamics of multiple infection and within-host competition in genetically diverse malaria infections. Am Nat.

[26] Vardo AM, Kaufhold KD, Schall JJ (2007). Experimental test for premunition in a lizard malaria parasite (*Plasmodium mexicanum*). J Parasitol.

[27] Voller A, Rossan R (1969). Immunological studies on simian malaria parasites IV. Heterologous superinfection of monkeys with chronic Plasmodium knowlesi infections. Trans Royal Soc Trop Med Hyg.

[28] Owusu-Agyei S, Binka F, Koram K, Anto F, Adjuik M, Nkrumah F (2002). Does radical cure of asymptomatic *Plasmodium falciparum* place adults in endemic areas at increased risk of recurrent symptomatic malaria?. Trop Med Int Health TM IH.

[29] Müller D, Charlwood J, Felger I, Ferreira C, do Rosario V, Smith T (2001). Prospective risk of morbidity in relation to multiplicity of infection with *Plasmodium falciparum* in S$$\tilde{a}$$o Tomé. Acta Tropica.

[30] Al-Yaman F, Genton B, Reeder J, Anders R, Smith T, Alpers M (1997). Reduced risk of clinical malaria in children infected with multiple clones of *Plasmodium falciparum* in a highly endemic area: a prospective community study. Trans Royal Soc Trop Med Hygiene.

[31] Smith T, Felger I, Tanner M, Beck HP (1999). 11. Premunition in *Plasmodium falciparum* infection: insights from the epidemiology of multiple infections. Trans Royal Soc Trop Med Hyg.

[32] McKenzie FE, Smith DL, O’Meara WP, Riley EM (2008). Strain theory of malaria: the first 50 years. Adv Parasitol.

[33] Collins WE, Jeffery GM (1999). A retrospective examination of the patterns of recrudescence in patients infected with *Plasmodium falciparum*. Am J Trop Med Hyg.

