# Supplementary material for: Asymptomatic Plasmodium falciparum infections may not be shortened by acquired immunity
Source: Malar J. 2015 Aug 4;14:294. doi: 10.1186/s12936-015-0813-1 (PMC4523025; doi:10.1186/s12936-015-0813-1)
Supplement: Additional file 1: — This document contains additional interpretations, analyses and figures based on the results of fitting all four candidate immigration-death models to the long-interval data. The implications of the estimated distributions of infection duration on the time to elimination, on the variation in infection durations and on senescence of infections are briefly discussed. [file 12936_2015_813_MOESM1_ESM.pdf]

Asymptomatic Plasmodium falciparum infections may not be shortened by acquired immunity

Michael T Bretscher[1,2], Nicolas Maire[1,2], Ingrid Felger[1,2], Seth Owusu-Agyei[3], Tom Smith[1,2]

[1]Swiss TPH, Basel, Switzerland
[2]University of Basel, Basel, Switzerland
[3]Kintampo Health Research Center, Kintampo, Ghana

Supporting Information

This document contains additional interpretations, analyses and figures based on the results of fitting all four candidate immigration-death models to the long-interval data. The implications of the estimated distributions of infection duration on the time to elimination, on the variation in infection durations and on senescence of infections are briefly discussed.

All distribution estimates

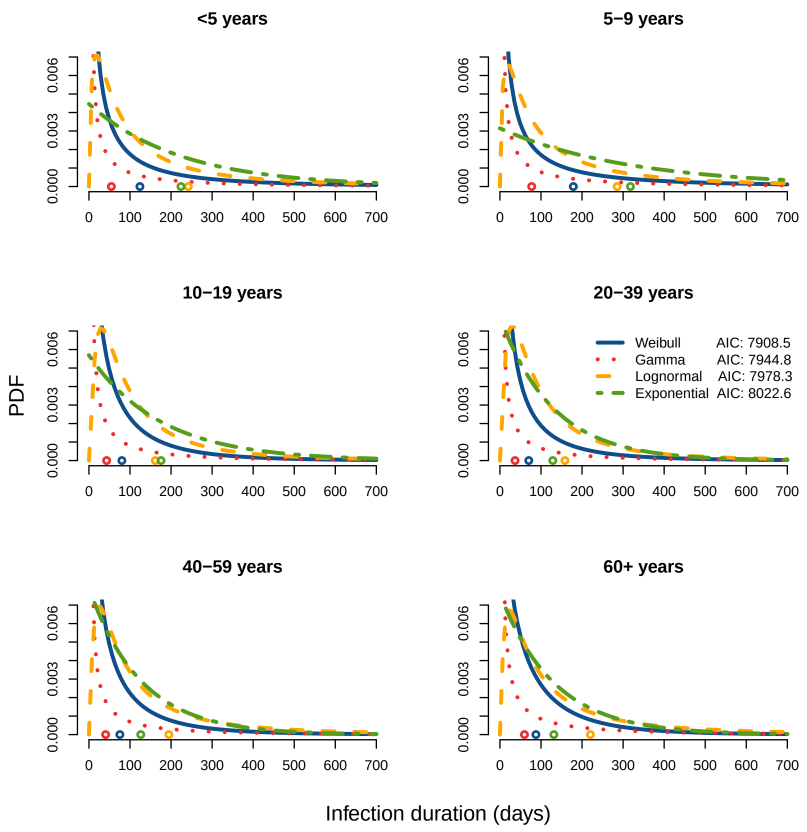

Figure 1: **Distribution of infection durations** - Shown are the distribution estimates of all clearance models. Of those, the exponential does not have any flexibility in shape, and the Lognormal is constrained to have a peak.

Standard deviation of infection durations

The standard deviation of infection durations, as estimated by the non-exponential models, is considerably smaller than suggested by the exponential model (Figure 2). Since the standard deviation of an exponential distribution is always equal to the mean it is likely that exponential models overestimate the variation of infection durations. All models show a peak in the standard deviation in the 5-9 year olds, very similar to the estimates of the mean duration.

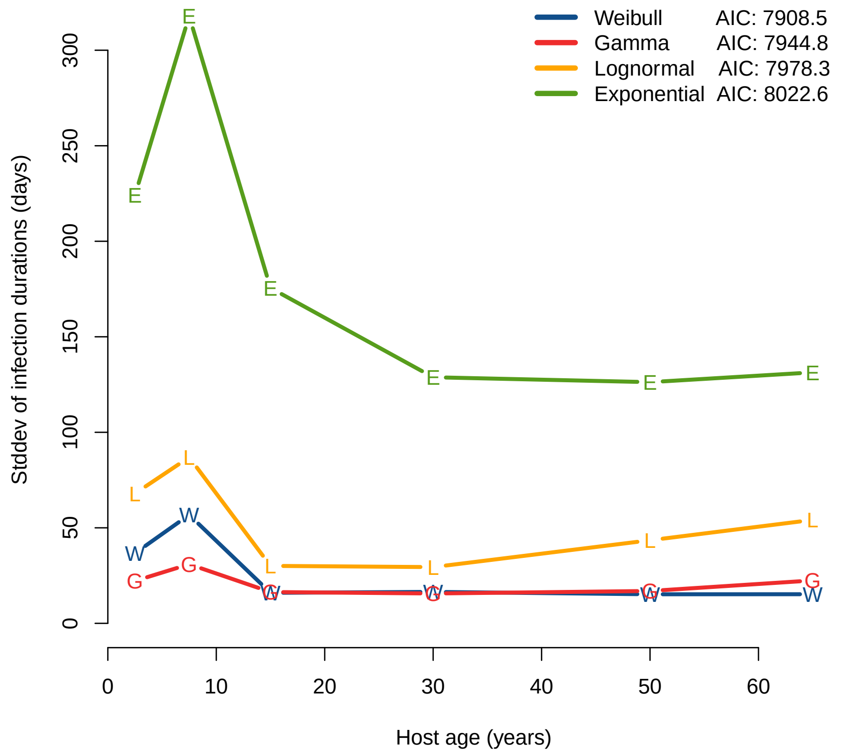

Figure 2: **Variation in the duration of infection** - The standard deviation of infection durations is plotted against the midpoint of each age group. While in an exponential distribution, the standard deviation is always equal to the mean, the other distributions have greater flexibility.

Mean resdidual lifetime of infections

The mean residual lifetime (**MRL**) is the additional time an infection is expected to last [1]. This quantity may change with the age of an infection, depending on the properties of the distribution of infection durations. It can be calculated from the shape and scale parameters as $\frac{1}{S(\xi)} \int_0^\infty t\, f(t+\xi)\; dt$, where ξ is the current age of an infection and S and f are the survivor function and PDF, respectively, of the corresponding survival model. MRL is plotted against age of infection in Figure 3. Only estimates from the best-fitting Weibull model and the worst-fitting exponential model are shown. The MRLs of the exponential estimates appear as horizontal straight lines because under an exponential model, clearance is independent of infection age. Weibull estimates, conversely, show an increase of MRL with the age of an infection. This effect is strongest in the first days after inoculation, and in the younger host ages.

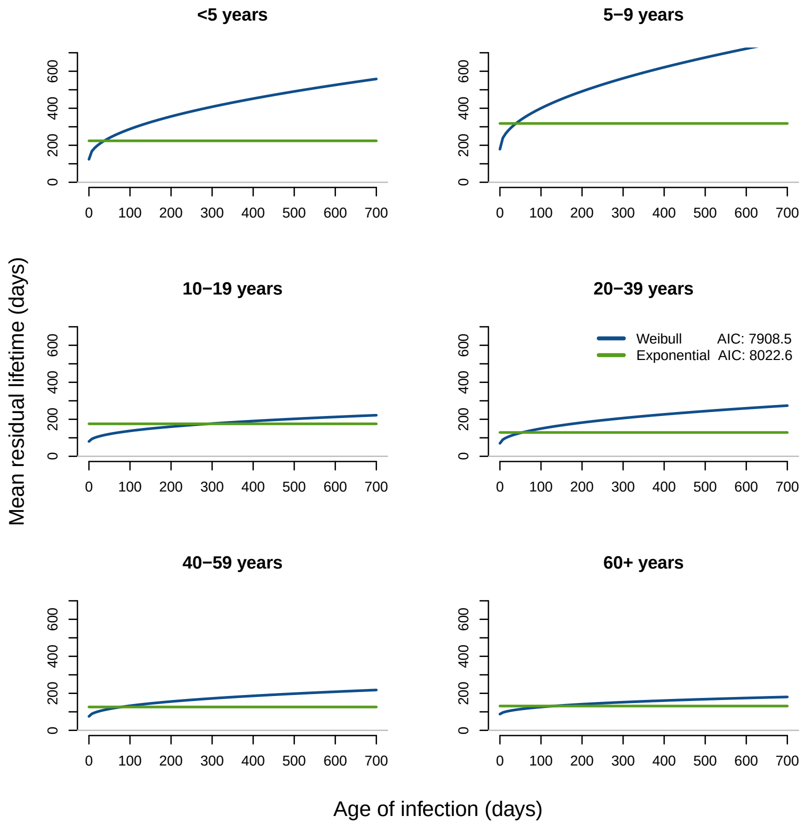

Figure 3: **Mean residual lifetime** - The mean residual lifetime (MRL) is the additional time an infection is expected to last. It depends on the current age of an infection and the distribution of infection durations. MRL is plotted against age of infection separately for each host age group.

Persistence of infections

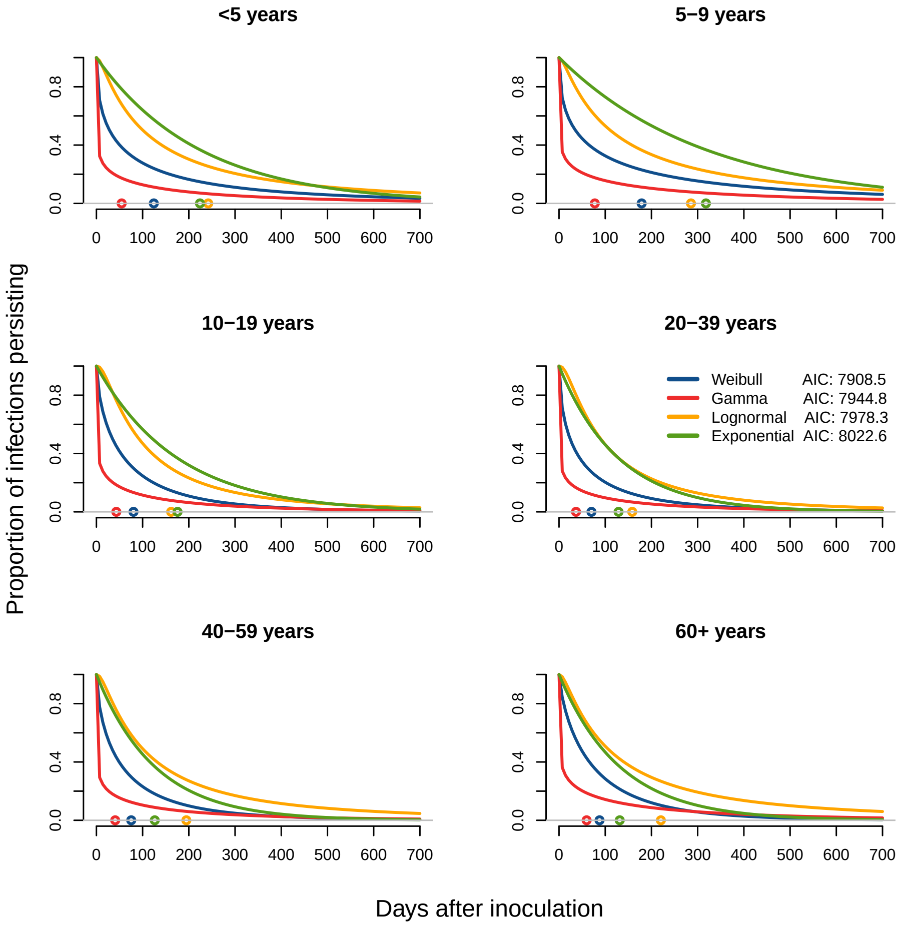

Figure 4: **Persistence of infections** - The proportion of clonal infections surviving is plotted against time after inoculation, for different models of infection clearance and across host age groups. Mean infection durations are indicated as circles on the abscissa.

Time to near-elimination

The discussion about local elimination or global eradication of malaria is again on the table, and the waiting time until the last infection is cleared is of considerable interest in this debate. However, estimates of the expected waiting time until 99% or 99.9% of infections are cleared, obtained from a distribution, do not form a complete theory of elimination or eradication: the size of the human population, the MOI, the intensity of residual transmission, the number of people at risk of infection, the intensity of surveillance through the health system and the rate of immigration of cases from elsewhere need to be considered as well. The distribution of the durations of clonal infections does, however, form part of such a theory, as presented by [2]. Smith *et al* consider "senescence" of infections, meaning that the rate at which infections are cleared (the hazard) increases with their age. Senescence of infections appeared to decrease the time to elimination in those simulations. A clearance rate that decreases with the age of infection should therefore prolong the time needed until near-elimination. Our results show that the choice of the statistical model influences estimates of time to near-elimination, identify the age group of 5-9 year olds as important target group for surveillance of activities during a potential elimination campaign.

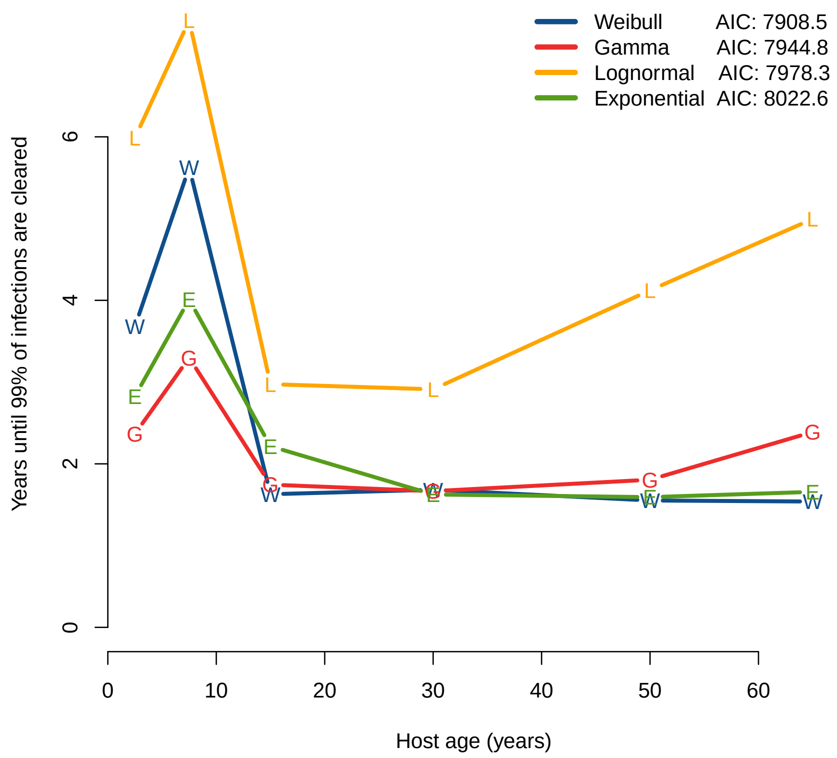

Figure 5: **Time until most infections are cleared** - The time is shown until 99% of infections are cleared after complete interruption of transmission. All models agree that the time required is longest in the age group of 5-9 years.

References

[1] Guess F, Proschan F: **Mean Residual Life: Theory and Applications**. *Quality Control and Reliability* 1988, :215.

[2] Smith D, Hay S: **Endemicity response timelines for Plasmodium falciparum elimination**. *Malaria Journal* 2009, **8**:87.
